# Comparison of SVM, RF and ELM on an Electronic Nose for the Intelligent Evaluation of Paraffin Samples

**DOI:** 10.3390/s18010285

**Published:** 2018-01-18

**Authors:** Hong Men, Songlin Fu, Jialin Yang, Meiqi Cheng, Yan Shi, Jingjing Liu

**Affiliations:** School of Automation Engineering, Northeast Electric Power University, Jilin 132012, China; menhong@neepu.edu.cn (H.M.); 2201500474@neepu.edu.cn (S.F.); 2201400444@neepu.edu.cn (J.Y.); 2201600376@neepu.edu.cn (M.C.); 2201500430@neepu.edu.cn (Y.S.)

**Keywords:** paraffin, paraffin odor analysis system, level, classify, grade

## Abstract

Paraffin odor intensity is an important quality indicator when a paraffin inspection is performed. Currently, paraffin odor level assessment is mainly dependent on an artificial sensory evaluation. In this paper, we developed a paraffin odor analysis system to classify and grade four kinds of paraffin samples. The original feature set was optimized using Principal Component Analysis (PCA) and Partial Least Squares (PLS). Support Vector Machine (SVM), Random Forest (RF), and Extreme Learning Machine (ELM) were applied to three different feature data sets for classification and level assessment of paraffin. For classification, the model based on SVM, with an accuracy rate of 100%, was superior to that based on RF, with an accuracy rate of 98.33–100%, and ELM, with an accuracy rate of 98.01–100%. For level assessment, the R^2^ related to the training set was above 0.97 and the R^2^ related to the test set was above 0.87. Through comprehensive comparison, the generalization of the model based on ELM was superior to those based on SVM and RF. The scoring errors for the three models were 0.0016–0.3494, lower than the error of 0.5–1.0 measured by industry standard experts, meaning these methods have a higher prediction accuracy for scoring paraffin level.

## 1. Introduction

China is a major paraffin producer, exporter, and consumer, annually exporting about 70% of total world paraffin trade volume, and playing an important role in the development of the international paraffin market. Paraffin is used as a packaging material, and in fiber board, rubber, insulation materials, and in other industries. The huge market requires the faster and more precise division of the different paraffin levels [1,2,3]. When the quality inspection calibration is performed on the post-factory paraffin products, odor intensity is one of the important quality indicators. Generally, the higher the quality of paraffin, the lower the odor concentration [4]. The Chinese petrochemical industry standard “Test Method for Paraffin Odor (SHT0414-2004)” is the most common paraffin odor identification method. This method involves the expert evaluation of odor using smell at the site. The paraffin odors are divided into five grades, from zero to four. The odor test group should consist of at least five experts, and each expert assigns an odor intensity value, then all values are averaged to obtain the final sample odor value. If the error is above 1.0, the evaluation should be repeated. The disadvantaged of expert sensory evaluation include the human resources requirement, lower precision, poor objectivity, and endangering evaluator health as a result of long-term exposure [5]. Therefore, having a rapid method for evaluating the paraffin odor level has practical significance for improving the profit margin in the paraffin economy and the product’s secondary development [6,7,8].

The electronic nose is a simple to operate, time-saving, and higher-precision smart bionic device that has been widely applied in the food science, environmental protection, and chemical industries [9,10,11,12]. For food science, Dymerski et al. [13] used Principal Component Analysis (PCA), Linear Discriminant Analysis (LDA), and Cluster Analysis (CA) with the furthest neighbor method to classify honey types. In another study, Banerjee et al. [14] applied sensor fusion to the classification and prediction of tea in quality assessments and achieved highly successful results. Valdez and Gutiérrez [15] employed PCA and Artificial Neural Network (ANN) to classify chocolate, based on an electronic nose with a pressure controlled canister. Aleixandre et al. [16] classified different ripening red and white grapes using electronic nose data. A high success rate was also achieved in the classification of drip and canned coffee [17]. For environmental protection, Ferreiro-González et al. [18] used several ignitable liquids, including gasoline, diesel, citronella, kerosene, and paraffin, on different substrates, including wood, cotton, cork, paper, and paperboard, to simulate distinguishing post-burn samples with an electronic nose. This method can be used as a green technology for fire debris analysis. In another study, Szulczyński et al. [19] used an electronic nose with semiconductor sensors and a PID-type sensor to distinguish odor interactions. Conner et al. [20] used an electronic nose to determine the existence of accelerants in specific areas. In the chemical industry, Feldhoff et al. [21] used a FOX 4000 electronic nose to examine the gas phase of about 20 diesel fuels. Gobbi et al. [22] rapidly diagnosed vegetable soups with an electronic nose. Berkhout et al. [23] analyzed the fecal volatile organic compound (VOC) profiles, in different sampling conditions and environments, with an electronic nose. Herrero et al. [24] developed a portable water quality pollution online detector based on an electronic nose that could classify a water sample blended with common pollutants (blank water, acetone, toluene, ammonia, formaldehyde, hydrogen peroxide, ethanol, benzene, dichloromethane, acetic acid, xylene, and dimethylacetamide), with an accuracy rate as high as 94%. Previous studies have showed that the electronic nose is widely used in many fields and can replace artificial sensory evaluation. Therefore, finding a method based on an electronic nose to classify and grade paraffin into different levels is necessary.

To improve the performance of the paraffin odor analysis system, many methods have been proposed [25]. Our focus was on a feature selection method which is an important aspect of machine learning. The multi-sensor fusion can replace artificial sensory evaluation, but also results in high-dimensionality and redundancy [26].Thus feature selection methods that find some features to represent the original feature set, have become an essential pre-processing step for developing the paraffin odor analysis system. Many feature selection methods have been used for the electronic nose, such as PCA [27,28], LDA [29], analysis of variance (ANOVA) [30], and multidimensional projection techniques [31].

In this paper, the paraffin odor analysis system was developed to test four kinds of paraffin samples with different odor levels. The PCA and Partial Least Squares (PLS) methods were used to reduce the number of dimensions of the original feature variables. Support Vector Machine (SVM), Random Forest (RF), and Extreme Learning Machine (ELM) were applied to three different feature data sets (#PCA, #PLS, and #Complete) to classify and grade paraffin samples with different odor levels.

## 2. Materials and Methods

### 2.1. Materials

In the experiments, four different kinds of paraffin were used with different odor levels, with scores of 0.6, 1.0, 1.2, and 1.5 provided by the same manufacturer. The paraffin score was obtained by experts from the Sinopec Fushun Research Institute of Petroleum and Petrochemicals. Each sample was sealed in single plastic bag to prevent cross contamination.

### 2.2. Sample Preparation 

To prevent the pollution of the paraffin odor during handling, disposable gloves were used when preparing the samples. Once the paraffin sample was dispensed, the gloves and scraper were replaced. The samples were prepared using the following methods:

(1) Cut the paraffin sample to be tested with a sharp scraper, obtain a non-adhesive sheet-shaped sample (about 0.5 mm).
(2) Accurately weigh-out 20.00 g of sheet paraffin and place it into a 500 mL customized glass bottle and seal it with a cover, letting the sample stand for 20 min for later use.

The sensor gas chamber temperature and relative humidity were controlled at 20 ± 2 °C and 65 ± 5% respectively. Twelve samples were prepared for each grade of paraffin, sequentially measured as a group according to the ascending order of paraffin odor grade (0.6-1.0-1.2-1.5). Twelve groups were measured and 48 groups of data were obtained. After the signals were pre-processed, steady voltage for 160 s was selected as the sample eigenvalue, then 48 × 16 dimensions of characteristic matrix **X** were obtained.

### 2.3. Design and Development of Paraffin Odor Analysis System

**Figure 1:**
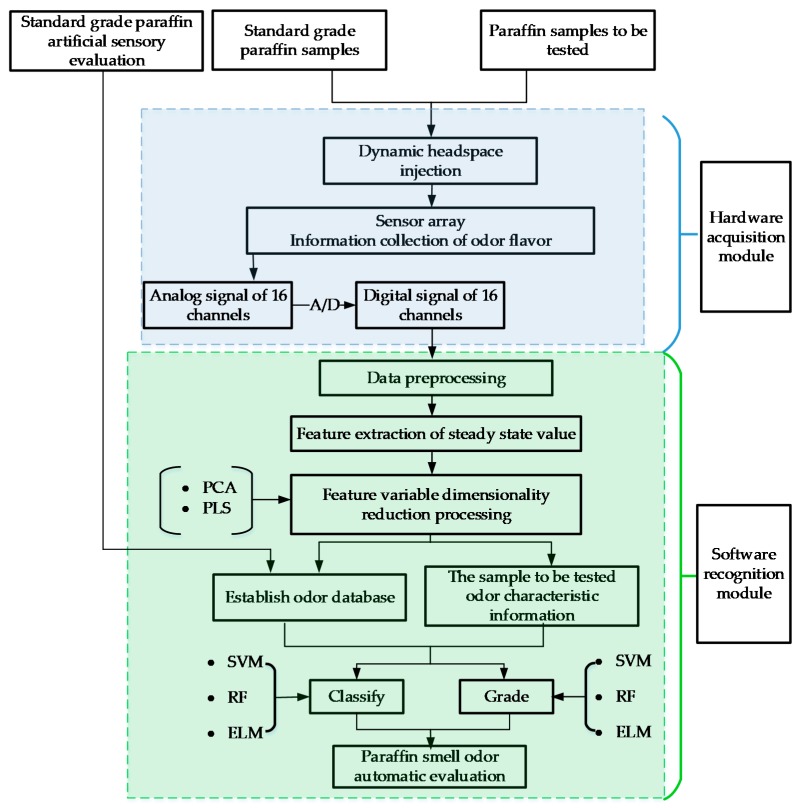
A schematic diagram of the paraffin odor analysis system.

The paraffin odor analysis system used in the experiments was designed and built in-house at the Northeast Electric Power University. This analysis system includes a headspace sampling device, a gas path part, a gas sensor array, a data acquisition unit, and a software analysis system. Figure 1 shows a schematic diagram of the paraffin odor analysis system. 

The system uses headspace gas sampling to inhale the headspace volatile gas with a built-in gas pump into the gas chamber, and an activated carbon purification device was set at the gas inlet. The sensor gas chamber and gas path are equipped with polytetrafluoroethylene (PTEE) thin films and conduits. The sensor array is composed of 16 gas sensors, which were selected according to the paraffin odor features. The sensors were: MQ-2, MQ-3, MQ-135, MQ-137, MQ-138, TGS-813, TGS-821, TGS-822, TGS-825, TGS-830, TGS-831, TGS-832, TGS-861, TGS-2610, TGS-2611, and 2m009.

**Figure 2:**
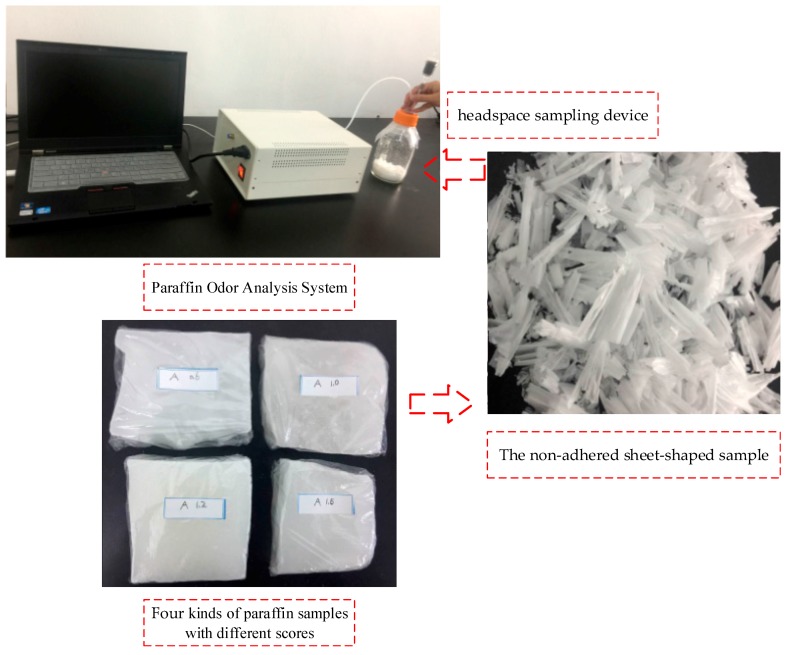
Paraffin odor analysis system.

**Figure 3:**
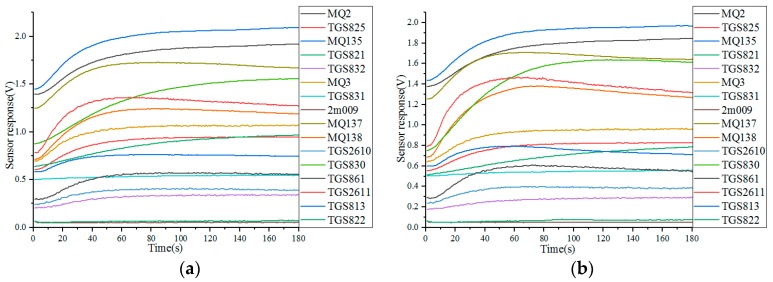

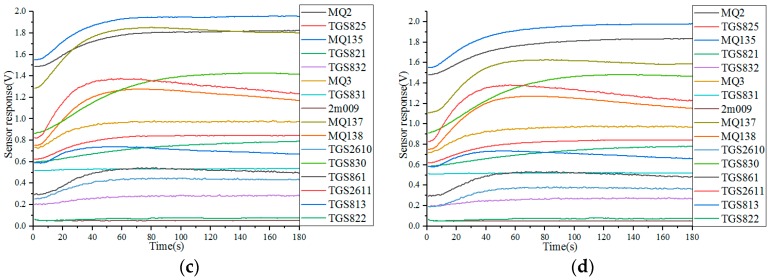
Sensor responses of four different paraffin samples: (**a**) Paraffin score 0.6; (**b**) Paraffin score 1.0; (**c**) Paraffin score 1.2; (**d**) Paraffin score 1.5.

The system software compiled on the development platform LabVIEW controlled the sample injection and cleaning. The system software can powerfully process data, and the imbedded MATLAB, as an invoking tool, pre-processed, analyzed and displayed the system data. Figure 2 shows the paraffin odor analysis system, and Figure 3 shows the sensor responses of four different paraffin samples.

### 2.4. Data Processing and Analysis Methods

Data processing and analysis included data pre-processing, feature extraction, variable selection, and mode recognition. 

#### 2.4.1. Variable Selection Method

Using multi-sensor fusion detection, a more complete odor “fingerprint” of the sample was obtained, but redundant information was still included to a certain extent. Before modeling, it is necessary to perform optimized selection on the variables, remove the redundant independent variables, and select the independent variables that can best reflect the input and output relationship to include in the modeling. In this paper, we selected PCA and PLS to reduce dimensions.

##### Principal Component Analysis (PCA)

PCA is a multivariate statistical method that uses a few indicators to replace the original variables by converting the multiple original indicators into several comprehensive indicators, using a dimension reduction processing technology [32,33]. The goal of optimizing the dimensionality reduction is to reduce the *n* sets of vectors to *k* dimensions (0 < *k* < *n*). Under orthogonal constraints, the maximum *k*-variance is used as a basis for the new variables. Using this method, the dimensions of the paraffin odor characteristic data set **X** were reduced to extract the main characteristics of the paraffin odor response.

Data set *X*, collected from the sensor array, was transposed and zero-equalized. The covariance matrix **D** of Matrix **X***^T^* was calculated by:

(1) $$D=\frac{1}{48}X^{T}X$$

Then, the **D** feature value *λ_i_*(*i =* 1, 2, …, *n*) and corresponding feature vector were obtained, and the feature vectors were input into Matrix *P* in descending corresponding feature value. The paraffin odor response feature matrix **W**, after being processed by principal component analysis, met the formula:

(2) $$W^{T}={\mathrm{PX}}^{T}$$

Calculate the variance contribution rate and the cumulative contribution rate:

(3) $$\alpha_{i}=\frac{\lambda_{i}}{\sum_{j=1}^{n}\lambda_{j}}$$

(4) $$\beta_{i}=\frac{\sum_{i=1}^{k}\lambda_{i}}{\sum_{j=1}^{n}\lambda_{j}}$$

where $\alpha_{i}$ is the variance contribution of *i* principal component, and $\beta_{j}$ is the cumulative contributions of the first *j* principal component’s variance.

##### Partial Least Squares (PLS)

Using PLS, the data information in the system were decomposed and screened to extract the aggregate variable that can best describe dependent variable. PLS is of uniquely advantageous in reducing data dimensions [34].

Through cross validation, the contribution margin to the prediction model accuracy of component *t* was measured to determine the effective components after dimensionality was reduced by PLS. The experiment determined whether adding a new component can improve the prediction function of the model. Let the data of the *i*th sample point be *X_i_* and ${\hat{y}}_{h\left(-i\right)}$ be the deleted sample point *i* (*i* = 1, 2, …, *n*) at the time of modeling. After *h* components were modeled, this model was used to calculate fitted value of *y_i_* to obtain the predicted error sum of squares of *y*, as shown in Equation (3). Additionally, all sample points were used to fit the regression equation with *h* components. Let the predicted value of the *i*th sample point be ${\hat{y}}_{hi}$, then the error sum of squares of *y* is as shown in Equation (4):

(5) $$PRESS_{h}={\sum_{i=1}^{n}\left(y_{i}-{\hat{y}}_{h(-i)}\right)}^{2}$$

(6) $$SS_{h}={\sum_{i=1}^{n}\left(y_{i}-{\hat{y}}_{hi}\right)}^{2}$$

where *SS_h_*_−1_ is the fitting error of equation with *h* − 1 components. For *PRESS_h_*, a component *t_h_* was added, but it contained the agitation error of the sample points. If the agitation error of the regression equation with *h* components is less than *h* − 1 to certain degree, then if a component *t_h_* is added, the predicted value will increase. Therefore, we hope $\left(PRESS_{h}/SS_{h-1}\right)\leq 0.95^{2}$. The cross-validation discrimination function is as shown in Equation (7):

(7) $$Q_{h}^{2}=1-\frac{PRESS_{h}}{SS_{h-1}}$$

Component *t_h_* cross validation $Q_{h}^{2}<0.0975$ indicates the realization of the target precision.

#### 2.4.2. Research Method

##### Support Vector Machine (SVM)

SVM is a supervised learning model that analyzes data and recognizes patterns; it can perform model classification and regression analysis [35,36,37]. One sample in a SVM model represents one point in space. As an effective and high-precision classification method, SVM was put forward by Cortes and Vapnik based on statistical learning theory [38]. The concrete implementation steps are as follows.

(1) SVM usually uses the following minimization optimization model to determine the regression function:
  (8) $$\min\frac{1}{2}{\left\|w\right\|}^{2}+c\sum_{i=1}^{m}\left(\xi_{i}^{*}+\xi_{i}\right)$$
  (9) $$s.t.\left\{ \begin{array}{l} y_{i}-w\cdot \zeta \left(x\right)-b\leq \varepsilon +\xi_{i}^{*}\\ \left(w\cdot \zeta \left(x\right)\right)+b-y_{i}\leq \varepsilon +\xi_{i}\\ \xi_{i}^{*},\xi_{i}\geq 0\left(i=1,2,\ldots ,m\right) \end{array}\right.$$
  where *w* is the weight vector, $\frac{1}{2}{\left\|w\right\|}^{2}$ is the expression of model complexity, *c* is the penalty factor, $\xi_{i}^{*}$ and $\xi_{i}$ are the relaxation factors, ζ(x) is a nonlinear transformation that maps data to high dimensional space, *b* is offset, and ε is the upper limit of error.
(2) The Lagrange multipliers $\alpha_{i}$ and $\alpha_{i}^{*}$ are introduced. The optimization model shown in Equations (10) and (11) can be transformed into the following dual optimization problem:
  (10) $$\max-\frac{1}{2}\sum_{i,j=1}^{m}\left(\alpha_{i}^{*}-\alpha_{i}\right)\left(\alpha_{j}^{*}-\alpha_{j}\right)k\left(X_{i},X\right)+\sum_{i=1}^{m}\alpha_{i}^{*}\left(y_{i}-\varepsilon \right)-\sum_{i=1}^{m}\alpha_{i}\left(y_{i}-\varepsilon \right)$$
  (11) $$s.t.\left\{ \begin{array}{l} \sum_{i=1}^{m}\alpha_{i}=\sum_{i=1}^{m}\alpha_{i}^{*}\\ 0\leq \alpha_{i},\alpha_{i}^{*}\leq c\left(i=1,2,\ldots ,m\right) \end{array}\right.$$
(3) The SVM regression function is obtained by solving the above problems:
  (12) $$f\left(x\right)=\sum_{i=1}^{m}\left(\alpha_{i}-\alpha_{i}^{*}\right)k\left(X_{i},X\right)+b$$
  (13) $$k\left(x_{i},x_{j}\right)=\exp\left(-\frac{{\left\|x_{i}-x_{j}\right\|}^{2}}{2\sigma^{2}}\right)=\exp\left(-\gamma {\left\|x_{i}-x_{j}\right\|}^{2}\right),\gamma >0$$

Two parameters are involved in the SVM calculation, namely penalty factor *c* and kernel parameter γ. This paper used the grid search method for optimization.

##### Random Forest (RF)

The RF algorithm was first put forward by Kam in 1995 [39]. RF is widely used for practical applications [40,41]. The decision tree can be rapidly built; therefore, training of hundreds of decision trees is even faster than training an artificial neural network. This algorithm is similar to Bagging; both of them perform resampling based on Bootstrap to generate multiple training sets. Conversely, RF randomly selects the split property set to build a decision tree. The detailed forest random algorithm process is as follows:

(1) Resampling is performed by Bootstrap to randomly generate *T* training sets *S*_1_, *S*_2_, …, *S_T_*.
(2) The corresponding decision tree *C*_1_, *C*_2_, …, *C_T_* for each training set is generated. Before a property is selected on the internal node, *m* properties are randomly selected from *M* properties as the split property set of the current node (*m* < *M*). Generally speaking, the *m* value is stable during the overall forest development process.
(3) Each tree is in complete development, pruning is not performed.
(4) For test set sample *X*, a test is performed by using each decision tree to obtain the corresponding class *C*_1_(*X*), *C*_2_(*X*), …, *C_T_*(*X*).
(5) By voting, the individual in *T* decision trees with the most outputs is selected as the test set sample *X*, then, the prediction is finished.

##### Extreme Learning Machine (ELM)

ELM is a rapid learning algorithm with a feedforward neural network with a single hidden layer that minimizes training error and obtains the minimum weight norm with good generalization performance and a high running speed [42]. The only free parameter studied in this algorithm is the connecting coefficient (or weight coefficient) between the hidden layer and the output layer. Based on the parameter, the linear parameter model can be built and the linear system can be solved [43].

At the beginning of training, ELM randomly generates *w* and *b*. Only by determining the number of neurons in the hidden layer and the infinitely differentiable activation function, can *β* be calculated. The ELM algorithm has the following steps:

(1) Set *N* different samples (*x_i_*, *t_i_*) ∈ *R^n×m^* and the activation function; the activation function of the neurons in the hidden layer is *g*(*x*):
  (14) $$\sum_{i=1}^{N}\beta_{i}g_{i}\left(x_{j}\right)=\sum_{i=1}^{N}\beta_{i}g_{i}\left(w_{i}\cdot x_{j}+b_{i}\right)=o_{j},j=1,\ldots ,N$$
  where *N* is the number of neurons in the hidden layer; *w_i_* is the weight between the *i*th hidden node and the input node, *w_i_*= [*w_i_*_1_, *w_i_*_2_, …, *w_in_*]*^T^*; *β_i_* is the output weight between the *i*th hidden node and the input node, *β_i_*= [*β_i_*_1_, *β_i_*_2_, …, *β_im_*]*^T^*; and *b_i_* is the deviation among the hidden nodes of the *i*th layer.
(2) The activation function of the feedforward neural network of a standard single hidden layer *g*(*x*) can approximate the training sample with zero errors:
  (15) $$\sum_{j=1}^{N}\left\|o_{j}-t_{j}\right\|=0$$
  Namely, the existence of *β_i_*, *w_i_*, and *b_i_* makes:
  (16) $$\sum_{j=1}^{N}\beta_{i}g\left(w_{i}\cdot x_{j}+b_{j}\right)=t_{j},\;j=1,\ldots ,N$$
(3) The above *N* equations can be written as *Hβ* = *T*:
  (17) $$H\left(w_{1},\ldots ,w_{N},b_{1},\ldots ,b_{N},x_{1},\ldots ,x_{N}\right)=\left[\begin{array}{ccc} g\left(w_{1}\cdot x_{1}+b_{1}\right) & \cdots & g\left(w_{N}\cdot x_{1}+b_{1}\right)\\ \vdots & \cdots & \vdots \\ g\left(w_{1}\cdot x_{N}+b_{1}\right) & \cdots & g\left(w_{N}\cdot x_{N}+b_{1}\right) \end{array}\right],\;\;\beta ={\left[\begin{array}{c} \beta_{1}^{T}\\ \vdots \\ \beta_{N}^{T} \end{array}\right]}_{N\times M},\;T={\left[\begin{array}{c} T_{1}^{T}\\ \vdots \\ T_{N}^{T} \end{array}\right]}_{N\times M}$$
  where *H* is the output matrix of the hidden layer of the neural network and *β* is the output layer connection weight.
(4) To train the feedforward neural network of the single hidden layer, a specific *β_i_’* should be found and *w_i_’* can be obtained with the following formula:
  (18) $$\left\|H\left(w_{1}^{\prime },\ldots ,w_{N}^{\prime },b_{1}^{\prime },\ldots ,b_{N}^{\prime }\right)\beta -T\right\|=\underset{w_{i},b_{i},\beta }{\min}\left\|H\left(w_{1},\ldots ,w_{N},b_{1},\ldots ,b_{N}\right)\beta -T\right\|$$

## 3. Results and Discussion

### 3.1. Variable Selection Results

#### 3.1.1. Variable Selection Results Based on PCA

**Figure 4:**
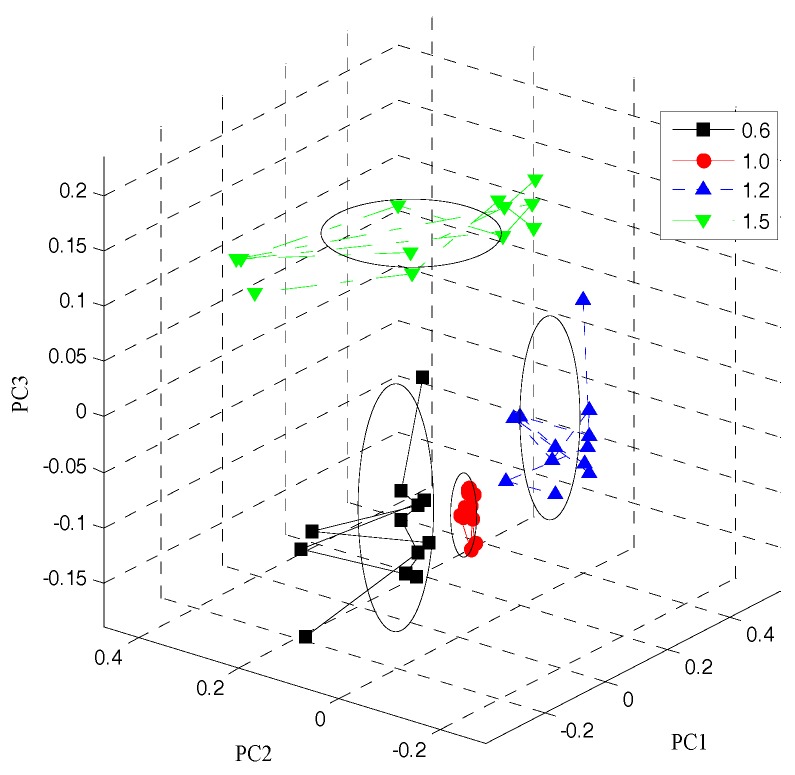
The PCA processing result.

The feature volatile gas data of the paraffin samples with different odor levels, collected from the gas sensor array, were analyzed with PCA. Figure 4 shows the paraffin sample PCA processing results for different odor levels. Under a standard environment, apparent differences exist among the volatile odor feature responses of the paraffin samples graded as 0.6, 1.0, 1.2, and 1.5.

The cumulative contribution rate of the principal components was 93.34%, representing all feature data. Finally, the first five principal components were extracted to form the new feature data set #PCA.

#### 3.1.2. Variable Selection Results Based on PLS

When the number of selected variables is as high as five, the system reached the target precision. Table 1 indicates the variable screening process.

Finally, five principal components were extracted from the original data set to form a new feature data set #PLS. Independent variable components are expressed as follows:

(19) $$\left\{ \begin{array}{l} t_{1}=-0.1126x_{1}-0.2221x_{2}+\ldots -0.2676x_{16}\\ t_{2}=-0.3708x_{1}-0.2415x_{2}+\ldots +0.3596x_{16}\\ t_{3}=-0.2145x_{1}-0.2161x_{2}+\ldots -0.3524x_{16}\\ t_{4}=-0.1108x_{1}-0.1807x_{2}+\ldots +0.2989x_{16}\\ t_{5}=+0.0041x_{1}-0.0776x_{2}+\ldots +0.5753x_{16} \end{array}\right.$$

### 3.2. Classification and Level Assessment of the Paraffin Samples

#### 3.2.1. Classification for the Paraffin Samples

##### Classification Based on SVM

**Figure 5:**
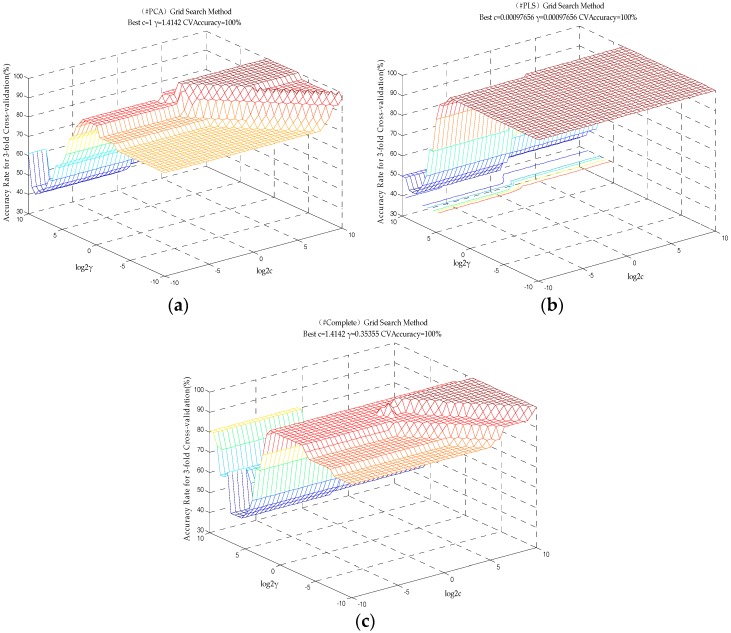
The Grid Search for the best parameter for constructing the LIBSVM model: (**a**) Based on the PCA-optimized feature set; (**b**) Based on the PLS-optimized feature set; (**c**) Based on the original feature set.

We randomly selected 36 groups of data as the training set, and the other 12 groups of data as the test set. The Grid Search for the best parameter for constructing the LIBSVM model is shown in Figure 5. In the (*c*, *γ*) grid point diagram, a higher accuracy rate is observed. The comparison of the classification results of# PCA, #PLS and #Complete combined with the SVM model are shown in Table 2.

As shown in Table 2, the prediction model training set, test set, and three-fold cross validation based on the PCA-optimized feature set, PLS-optimized feature set, and the original feature set were 100% accurate.

##### Classification Based on RF

The main parameters designed by RF are the value of the mtry and the number of decision trees. The default mtry is the square root of the total number of variables; hence, based on the variable dimension of the three feature sets, the system mtry was two, two, and four. The number of input neurons was 5, 5, and 16, representing the morphological feature of the nucleus of the corresponding number. The four output neurons indicate the grade of the paraffin sample: 0.6, 1.0, 1.2, and 1.5. Here, we only analyze and discuss the prediction model accuracy rate when the number of decision trees is 100. The accuracy rate of test set was 91.67–100%. The three feature sets (#PCA, #PLS and #Complete) combined with the RF model accurately classified the paraffin samples with different odor levels. To reduce the effect of randomness, 100 prediction models were built, then the accuracy rates were averaged to describe the classification average accuracy rate of the current model. We found that average accuracy rate of the test sets of the three feature sets was 99.58–100%, whereas the average accuracy rate of RF based on a PLS-optimized feature set was as high as 100%.

**Figure 6:**
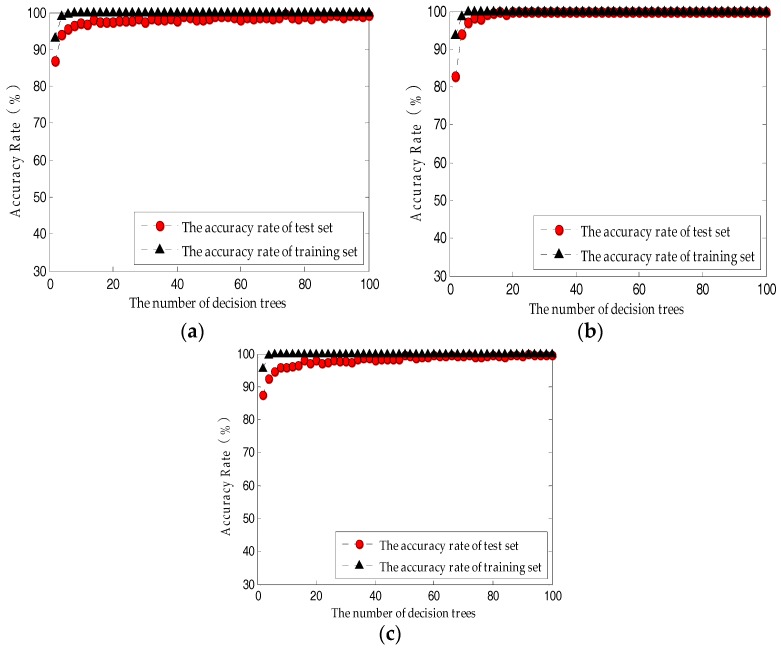
The influence of the number of decision trees on RF performance: (**a**) Based on the PCA-optimized feature set; (**b**) Based on the PLS-optimized feature set; (**c**) Based on the original feature set.

For the model based on the PLS-optimized feature set, when the number of decision trees was above 20, the average accuracy rate of either training set or test set was as high as 100% (Figure 6b). When the number of decision trees in the model continuously increased, the system was still stable. By comparing Figure 6b,c, the RF neural network model based on #PLS is superior to the model based on #Complete, which means that in this prediction system, the paraffin odor multi-sensor fusion detection results in certain information redundancy; the system stability can be enhanced by appropriately reducing the number of dimensions.

The above comparative study shows that the number of decision trees based on the RF model should be 66–100; the average accuracy rate of the model test set is 99.58–100%.

##### Classification Based on ELM

Paraffin samples of different odor levels were analyzed with ELM. To reduce the effects of randomness, 100 prediction models were built, then the accuracy rates were averaged to describe the classification average accuracy of the current model. The average accuracy rate of the test sets of the three feature sets was 98.83–100%. When the number of neurons in the hidden layer of the ELM model based on the PLS-optimized feature set was 100, the average accuracy rate of the test set was as high as 100%.

**Figure 7:**
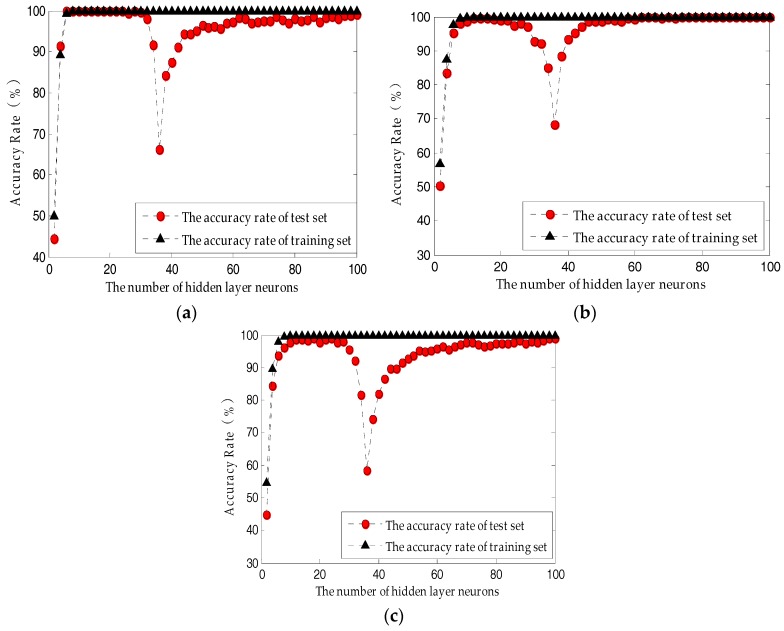
The influence of the number of hidden layer neurons on ELM performance: (**a**) Based on the PCA-optimized feature set; (**b**) Based on the PLS-optimized feature set; (**c**) Based on the original feature set.

When applying the paraffin recognizing model for different odor levels, the accuracy rate of the training set was as high as 100%, whereas this was not always the case for the test set, which means the ELM function was over trained, or the model generalization ability was weaker. As shown in Figure 7b, for the model based on the PLS-optimized feature set, when the number of nodes in the hidden layer was above 62, the average accuracy of either the training set or the test set was as high as 100%. As shown in Figure 7a, for the model based on the PCA-optimized feature set, when the number of hidden nodes was 12–20, the accuracy of either the training set or the test set was as high as 100%. By comparing Figure 7b,c, the prediction model based on #Complete is obviously inferior to that based on #PLS, which means the system stability and efficiency were enhanced by PLS’s reduction of the number of dimensions of the feature data. The above comparative study shows that by comprehensively considering the number of hidden neurons and the modeling speed, the number of neurons in the hidden layer based on the ELM network model should be 12–20 for the classification of paraffin’s different odor levels.

#### 3.2.2. Level Assessment for the Paraffin Samples

##### Level Assessment Based on SVM

As shown in Table 3, the R^2^ related to the training set and the test set of the model were above 0.98 and 0.89, respectively. By comparing the three groups of models, the R^2^ related to the model test set, based on the PCA-optimized and PLS-optimized feature sets, was R^2^ > 0.94, indicating the effect is superior to the model based on the original feature set.

The absolute value of the error range of the prediction model based on the three feature data sets (#PCA, #PLS, and #Complete) of the paraffin samples is shown in Table 4. The SVM network was used to predict the paraffin odor level score, with a score error of 0.0016–0.2163, which is lower than artificial grading error of 0.5–1.0, indicating this method can grade the level of the paraffin odor in industry production.

##### Level Assessment Based on RF

As shown in Table 5, the R^2^ related to the training set and the test set of the model were above 0.97 and 0.87, respectively. By comparing the three groups of models, the prediction models based on #PCA and #PLS (R^2^ > 0.8717 and R^2^ > 0.9645, respectively) were not optimal compared to that based on #Complete (R^2^ > 0.9896).

The absolute value of the error range of the prediction model based on the three feature data sets (#PCA, #PLS, and #Complete) of the paraffin samples with different odor levels is shown in Table 6. The prediction score error was 0.0024–0.3494, which is far lower than the artificial grading error of 0.5–1.0, indicating this method can grade the paraffin odor level in industry production.

##### Level Assessment Based on ELM

As shown in Table 7, the R^2^ related to the training set and the test set of the prediction model were both above 0.97. By comparing the three groups of feature sets, the index related to the model test set based on the PCA-optimized and PLS-optimized feature sets was superior to the prediction model based on the original feature set, whereas the prediction model based on the PLS-optimized feature set was optimal.

**Figure 8:**
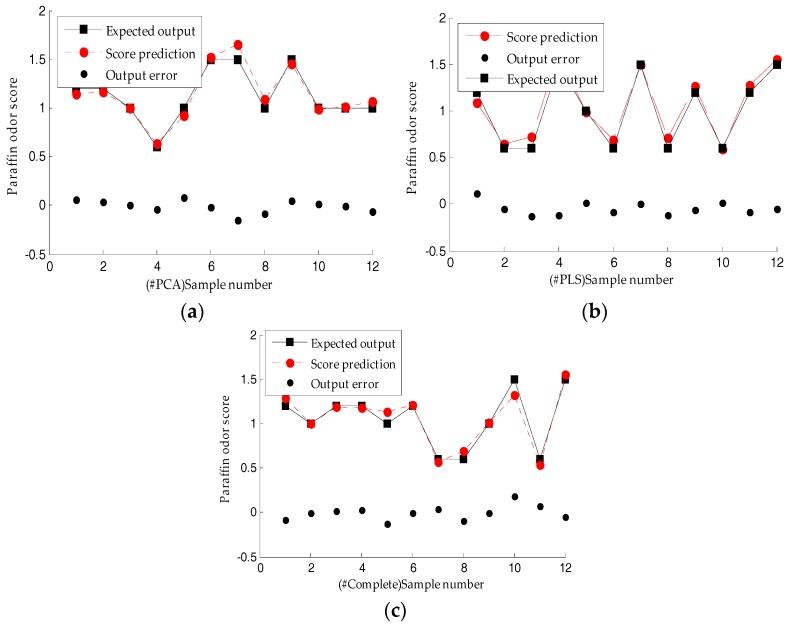
Score prediction of the paraffin odor based on the ELM model: (**a**) Based on the PCA-optimized feature set; (**b**) Based on the PLS-optimized feature set; (**c**) Based on the original feature set.

The paraffin sample prediction effects based on the ELM model are as shown in Figure 8. The absolute value of the error range of the prediction model based on the three feature data sets (#PCA, #PLS, and #Complete) of paraffin samples with different odor levels is shown in Table 8. The prediction score error was 0.0033–0.1804, which is far lower than the artificial grading error of 0.5–1.0, indicating this method can effectively grade the paraffin odor level in industry production.

## 4. Conclusions

(1) Design of paraffin odor analysis system: in this paper, we introduced a new method for testing paraffin odor level based on the electronic nose, designed and developed the paraffin odor analysis system. This system can analyze, screen, and recognize the paraffin odor feature response and grade the odor of an unknown paraffin sample.
(2) Classification of paraffin samples: SVM, RF, and ELM were applied to three different feature data sets to build the model and compare the model accuracy rate and regression parameters. By comprehensively comparing the three models, we found that during the classification of paraffin odor, the prediction model based on the SVM network, with an accuracy rate of 100%, was superior to the networks based on RF, with an accuracy rate of 98.33–100%, and ELM, with an accuracy rate of 98.01–100%.
(3) Level assessment of paraffin samples: during the recognition of the paraffin samples with different odor levels, the prediction models based on the three different feature sets were able to predict the score of the paraffin sample. The R^2^ related to the training set of the model was above 0.97 and the R^2^ related to test set was above 0.87. The paraffin odor level scores were predicted by three methods, SVM, RF, and ELM, and the predicted score error was 0.0016–0.3494, which is considerably lower than the 0.5–1.0 error measured by industry standard experts. Therefore, the three methods have higher prediction precision for paraffin odor level scores. By comprehensively comparing the relevant coefficients of the three models, the generalization of the model based on ELM was superior to that based on SVM and RF.

## Figures and Tables

**Table 1:** The feature screening process.

Time	Selected Variable	Cross-Validation Discrimination Function $Q_{h}^{2}$
1	t1	1
2	t2	0.7896
3	t3	0.2248
4	t4	0.1760
5	t5	−0.0638

**Table 2:** Comparison of classification results of #PCA, #PLS and #Complete combined with SVM model.

Feature Set	Best Parameter		Accuracy Rate for Training Set (%)	Accuracy Rate for 3-Fold Cross-Validation (%)	Accuracy Rate for Test Set (%)
	Penalty Factor *c*	Kernel Parameter *γ*			
#PCA	1	1.4142	100	100	100
#PLS	0.00097656	0.00087656	100	100	100
#Complete	1.4142	0.35355	100	100	100

**Table 3:** Comparison of the #PCA, #PLS, and #Complete parameters combined with the SVM model.

Feature Set	The Best Parameter		Training Set		Test Set	
	*c*	*γ*	R^2^	RMSE	R^2^	RMSE
#PCA	32	0.1767	0.9829	00481	0.9502	0.1376
#PLS	5.6596	0.125	0.9894	0.0491	0.9639	0.1968
#Complete	2.8284	0.0883	0.9974	0.0289	0.8913	0.1317

**Table 4:** Prediction error of different paraffin odor levels based on SVM.

Feature Set	Maximum Error	Minimum Error
#PCA	0.1448	0.0041
#PLS	0.2163	0.0044
#Complete	0.1690	0.0016

**Table 5:** Comparison of the #PCA, #PLS, and #Complete parameters combined with the RF model.

Feature Set	Training Set		Test Set	
	R^2^	RMSE	R^2^	RMSE
#PCA	0.9767	0.1951	0.8717	0.3707
#PLS	0.9869	0.1197	0.9645	0.2022
#Complete	0.9865	0.1089	0.9896	0.1537

**Table 6:** Prediction error of different paraffin odor levels based on RF.

Feature Set	Maximum Error	Minimum Error
#PCA	0.3494	0.0121
#PLS	0.1793	0.0024
#Complete	0.1266	0.0045

**Table 7:** Comparison of the #PCA, #PLS, and #Complete parameters with the ELM model.

Feature Set	Training Set		TEST SET	
	R^2^	RMSE	R^2^	RMSE
#PCA	0.9730	0.0727	0.9438	0.1437
#PLS	0.9972	0.0208	0.9675	0.1793
#Complete	0.9878	0.0472	0.9341	0.1741

**Table 8:** Prediction error of different paraffin odor levels based on RF.

Feature Set	Maximum Error	Minimum Error
#PCA	0.1487	0.0016
#PLS	0.1239	0.0061
#Complete	0.1804	0.0033
